# Parliament and the Profession

**Published:** 1915-11-13

**Authors:** 


### PARLIAMENT AND THE PROFESSION.

### The Army's and the People's Teeth.

A number of questions were asked in the House of Commons last week with regard to the dental treatment of soldiers and the enlistment of dentists by peacefully persuasive methods. Thus Mr. Butcher asked the Under-Secretary of State for War whether he was aware that in certain cases soldiers had been ordered by the medical officer to their unit to have all their teeth extracted, and that the War Office had refused to make them any allowance for the cost of replacing their teeth when extracted. The reply was to the effect that the War Office was not aware of any such refusal. With regard to the enlistment of dentists, Mr. Raffan asked whether, in view of the dental requirements of the community, practitioners in dentistry would be starred as men to be exempted from military service under Lord Derby's scheme of recruiting. Mr. Tennant replied that it was not intended that the facilities for consulting dentists should be arbitrarily curtailed. This is rather vague!

### Speculating in Drugs.

In the House of Commons on November 4 Commander Bellairs asked the President of the Board of Trade whether his attention had been drawn to the increase in the price of quinine, and whether he would cause inquiries to be made as to whether this rise had been brought about by actions of individuals who are exploiting the war. Mr. Pretyman replied that the advance had been under the consideration of the Government, and it was recently decided to prohibit the export of that commodity. Since that decision became known there had been a sharp fall in price. This sounds very plausible, but the action of the Government in placing an embargo on the export of quinine ought to have been taken several weeks ago. During the interval which elapsed between the time when America began to deplete our stocks and the date of the Order in Council the price of the drug had quadrupled and speculators had reaped a rich harvest. If our supplies had been properly conserved there is no reason why the price of this valuable drug should have advanced to anything like the extent of the recent fantastic increase, one of the effects of which was to impose a tax on hospitals the stocks of which had to be replenished. It is an extraordinary thing that the Government does not seek proper expert advice on these matters.

### What is a War Hospital Unit?

Several questions have recently been asked in the House of Commons with respect to the services of a hospital staff from Australia (The Hospital, October 23, p. 90, and October 30, p. 102). The matter was again alluded to last week, when Mr. Cathcart Wason asked the Under-Secretary of State for War what number of medical officers, sisters, and nurses constituted a unit; and if, in view of the fact that a large contingent of medical officers, sisters, and nurses had been here for weeks with nothing to do, he would state if there was a reasonable possibility of their being employed as a unit at Salonika or elsewhere. Mr. Tennant replied that a general hospital consisted of 34 officers, 72 nurses, and 201 non-commissioned officers and men, with about 1,000 tons of ordnance and medical stores. The *personnel* referred to by Mr. Wason are at the disposal of the Australian military authorities, and most of them are employed on special work in connection with the large number of Australian sick and wounded at present in the United Kingdom.

### The Future of National Health Insurance.

Of late many misleading statements have been made in the public Press with regard to the finances of the Insurance Act, and at least one member of Parliament, who ought to know better, has made speeches which have created the impression that in London they are verging upon bankruptcy. There is no substantial ground whatever for these misgivings, and the administrative head of the Insurance Act said as much in the House of Commons on November 4, in reply to a question by Sir J. D. Rees. It is, of course, common knowledge that in several areas the sums allotted for the sanatorium benefit are insufficient, and that in London it has been found necessary to propose that the deficit should be made up by transferring money from the Administration Fund to the Sanatorium Benefit Fund. This is a perfectly regular transaction, for which provision is made in the regulations, and any member of Parliament who bases on this proposal the argument that the Insurance Act is doomed to early failure is, to say the least, guilty of an error of judgment.

